# Individual differences *v.* the average patient: mapping the heterogeneity in ADHD using normative models

**DOI:** 10.1017/S0033291719000084

**Published:** 2019-02-14

**Authors:** Thomas Wolfers, Christian F. Beckmann, Martine Hoogman, Jan K. Buitelaar, Barbara Franke, Andre F. Marquand

**Affiliations:** 1 Department of Human Genetics, Donders Institute for Brain, Cognition and Behavior, Radboud University Medical Center, Nijmegen, The Netherlands; 2 Donders Centre for Cognitive Neuroimaging, Donders Institute for Brain, Cognition and Behavior, Radboud University, Nijmegen, The Netherlands; 3 Department of Cognitive Neuroscience, Donders Institute for Brain, Cognition and Behavior, Radboud University Medical Center, Nijmegen, The Netherlands; 4 Centre for Functional MRI of the Brain (FMRIB), University of Oxford, Oxford, UK; 5 Karakter Child and Adolescent Psychiatry University Center, Radboud University Medical Center, Nijmegen, The Netherlands; 6 Department of Psychiatry, Donders Institute for Brain, Cognition and Behavior, Radboud University Medical Center, Nijmegen, The Netherlands; 7 Department of Neuroimaging, Institute of Psychiatry, King's College London, London, UK

**Keywords:** Attention-deficit/hyperactivity disorder, heterogeneity, normative modeling, precision medicine

## Abstract

**Background:**

The present paper presents a fundamentally novel approach to model individual differences of persons with the same biologically heterogeneous mental disorder. Unlike prevalent case-control analyses, that assume a clear distinction between patient and control groups and thereby introducing the concept of an ‘average patient’, we describe each patient's biology individually, gaining insights into the different facets that characterize persistent attention-deficit/hyperactivity disorder (ADHD).

**Methods:**

Using a normative modeling approach, we mapped inter-individual differences in reference to normative structural brain changes across the lifespan to examine the degree to which case-control analyses disguise differences between individuals.

**Results:**

At the level of the individual, deviations from the normative model were frequent in persistent ADHD. However, the overlap of more than 2% between participants with ADHD was only observed in few brain loci. On average, participants with ADHD showed significantly reduced gray matter in the cerebellum and hippocampus compared to healthy individuals. While the case-control differences were in line with the literature on ADHD, individuals with ADHD only marginally reflected these group differences.

**Conclusions:**

Case-control comparisons, disguise inter-individual differences in brain biology in individuals with persistent ADHD. The present results show that the ‘average ADHD patient’ has limited informative value, providing the first evidence for the necessity to explore different biological facets of ADHD at the level of the individual and practical means to achieve this end.

## Introduction

Two patients suffering from the same mental disorder may show differences in symptom expression, behavior, and pathophysiology. Case-control research paradigms ignore these sources of heterogeneity; they assume that each diagnostic group is a distinct entity. A key goal in many such studies is to identify biological markers that are reliable indicators of disease state. However, markers identified through this approach generally explain only a small part of the variance linked to mental disorders (Schmaal *et al*., 2016; Hibar *et al*., 2017; Hoogman *et al*., 2017). Therefore, the case-control paradigm has been challenged in recent years. For example, large international initiatives aim to bridge the gap between a psychiatric diagnosis and its underlying biology through the integration of information across multiple dimensions (Insel, 2009; Insel *et al*., 2010; Schumann *et al*., 2014), yielding subgroups of patients stratified based on behavior (Fair *et al*., 2013; Mostert *et al*., 2015) or biological functioning (Marquand *et al*., 2016*b*). While such stratification approaches may produce more homogeneous diagnostic groups, these approaches still do not fully inform on how patients differ from one another in terms of the underlying biology. Therefore, inter-individual differences become a novel research focus (Foulkes and Blakemore, 2018; Seghier and Price, 2018).

Attention-deficit/hyperactivity disorder (ADHD) is a prevalent and impairing neurodevelopmental disorder, which persists into adulthood in a substantial part of the patients (Simon *et al*., 2009). Reliable group differences between healthy individuals and those with ADHD have been established for various biological readouts (Bush *et al*., 2005; Seidman *et al*., 2005; Valera *et al*., 2007; Cortese and Castellanos, 2012; van Ewijk *et al*., 2012; van Rooij *et al*., 2015; Wolfers *et al*., 2016). These include neuroimaging-based brain readouts, where differences in gray matter volume, white matter volume, as well as functional brain readouts (Frodl and Skokauskas, 2012; Onnink *et al*., 2014; Faraone *et al*., 2015; Greven *et al*., 2015; Wolfers *et al*., 2015*b*, 2017; Francx *et al*., 2016; Norman *et al*., 2016) have been reported. However, these differences are mostly of small to medium effect size and have not readily translated into individualized predictions (Wolfers *et al*., 2015*a*). In line with this observation, evidence accumulated in the last decades points towards ADHD being characterized by a high degree of heterogeneity (Faraone *et al*., 2015): More specifically, individuals with ADHD can differ from each other in their symptom profiles (clinical heterogeneity), their exposure to environmental stressors (environmental heterogeneity), and the underlying biology of their disorder (biological heterogeneity). This complexity, and the rather exclusive research focus on a categorical diagnosis, has hindered progress towards a better understanding of ADHD (Burmeister *et al*., 2008; Sullivan *et al*., 2012). Moreover, the developmental character of ADHD has been shown in numerous studies, and differences in brain development and aging have been observed across the lifespan (Shaw *et al*., 2007; Greven *et al*., 2015; Hoogman *et al*., 2017). Therefore, the importance of modeling ADHD across the lifespan has become increasingly apparent (Shaw *et al*., 2006; Hoogman *et al*., 2017). For example, individually different growth trajectories of different brain regions may be an important aspect of this complex phenotype (Shaw *et al*., 2006, 2007).

In this study, we aimed to quantify and map the brain structural heterogeneity in adults with persistent ADHD, at the level of the individual patient. We employed a normative modeling approach for this purpose, which provides a perspective that is fundamentally different from the classic case-control approach. A normative model can be understood as a statistical model that maps demographic, behavioral, or any other variable to -for example- a quantitative brain read-out (Marquand *et al*., 2016*a*), whilst providing estimates of centiles of variation within the population. Then, the individual can be placed within the normative range, allowing for the characterization of differences between individual patients in relation to the healthy range. In this way, we (i) chart the heterogeneity in abnormalities of brain structure at the level of the individual with ADHD, and (ii) investigate the degree of spatial overlap in terms of deviations from the normative model to provide concrete estimates for disorder heterogeneity. Based on previous case-control comparisons (e.g. Onnink *et al*., 2014; Faraone *et al*., 2015; Greven *et al*., 2015; Wolfers *et al*., 2015*b*, 2017; Francx *et al*., 2016), which introduced the notion of the ‘average ADHD patient’, we expected participants with ADHD to show on average larger negative deviations from the normative brain ageing model than healthy individuals. More importantly, we anticipated that the individual local deviance from the normative model would differ substantially between individuals, suggesting that previous group-level distinctions provide an incomplete picture of the neurobiological abnormalities in ADHD and disguise extreme inter-individual differences between individuals with ADHD.

## Methods

### Participants

We selected adult participants with persistent ADHD and healthy individuals from the Dutch cohort of the International Multicenter persistent ADHD CollaboraTion (IMpACT; Hoogman *et al*., 2011; Mostert *et al*., 2015), based on data availability for structural MRI images. Participants with persistent ADHD were recruited from the Department of Psychiatry of the Radboud University Medical Center and through advertisements. In this recruitment process, the participants with persistent ADHD were matched for gender, age, and estimated intelligence to a healthy individual population. All participants underwent psychiatric assessments, neuropsychological testing, and neuroimaging. The diagnostic interview for persistent ADHD (DIVA; Sandra Kooij *et al*., 2008) was conducted to confirm the diagnosis of ADHD in adulthood. This interview focuses on the 18 DSM-IV symptoms of ADHD and uses realistic examples to thoroughly investigate whether a symptom is currently present or was already present in childhood (Sandra Kooij *et al*., 2008). In all participants in the ADHD cohort, a childhood history of ADHD symptoms was established, and persistent ADHD was diagnosed. The ADHD Rating Scale-IV was filled in by each participant to report current symptoms of attention and hyperactivity/impulsivity (Pappas, 2006). To assess comorbidities, the structured clinical interviews (SCID-I and SCID-II) for DSM-IV were administered (van Groenestijn *et al*., 1999; Weertman *et al*., 2003; Lobbestael *et al*., 2011). The inclusion criteria for participants with ADHD were: (i) DSM-IV-TR criteria for ADHD met in childhood as well as in adulthood, (ii) no psychosis, (iii) no substance use disorder, (iv) full-scale intelligence estimate >70 (prorated from Block Design and Vocabulary of the Wechsler Adult Intelligence Scale-III (Wechsler, 2012), (v) no neurological disorders, (vi) no obvious sensorimotor disabilities, (vii) no medication use other than psychostimulants or atomoxetine. Additional inclusion criteria for healthy individuals were: (viii) no current neurological or mental disorder according to DIVA, SCID-I, or SCID-II, (ix) no first-degree relatives with ADHD or other major mental disorders. All participants were Dutch and of European Caucasian ancestry. This study was approved by the regional ethics committee (Centrale Commissie Mensgebonden Onderzoek: CMO Regio Arnhem – Nijmegen; Protocol number III.04.0403). Written informed consent was obtained from all participants.

### MRI acquisition

Whole brain imaging was performed using a 1.5 T scanner (Magnetom Avanto, Siemens Medical Systems) with a standard 8-channel head coil. A high-resolution T1-weighted magnetization-prepared rapid-acquisition gradient echo (MPRAGE) anatomic scan was obtained from each participant, in which the inversion time (TI) was chosen to provide optimal gray matter–white matter T1 contrast [repetition time (TR) 2730 ms, echo time (TE) 2.95 ms, TI 1000 ms, flip angle 7°, field of view (FOV) 256 × 256 × 176 mm^3^, voxel size 1.0 × 1.0 × 1.0 mm^3^]. The T1 images served as a basis for the extraction of gray and white matter volumes.

### Estimation of gray and white matter volume

Prior to gray matter volume estimation, all participants’ T1 images were rigidly aligned using statistical parametric mapping version 12 (SPM-12). Subsequently, images were segmented, normalized, and bias field–corrected using ‘new segment’ from SPM12 (http://www.fil.ion.ucl.ac.uk/spm; Ashburner and Friston, 2000, 2005) yielding images containing gray and white matter segments. We then used DARTEL (Ashburner, 2007) to create a study-specific gray matter template to which all segmented images were normalized. Subsequently, all gray matter volumes were smoothed with an 8-mm full width half maximum (FWHM) Gaussian kernel, and the normative model was estimated.

### Normative modeling

**Fig. 1.**
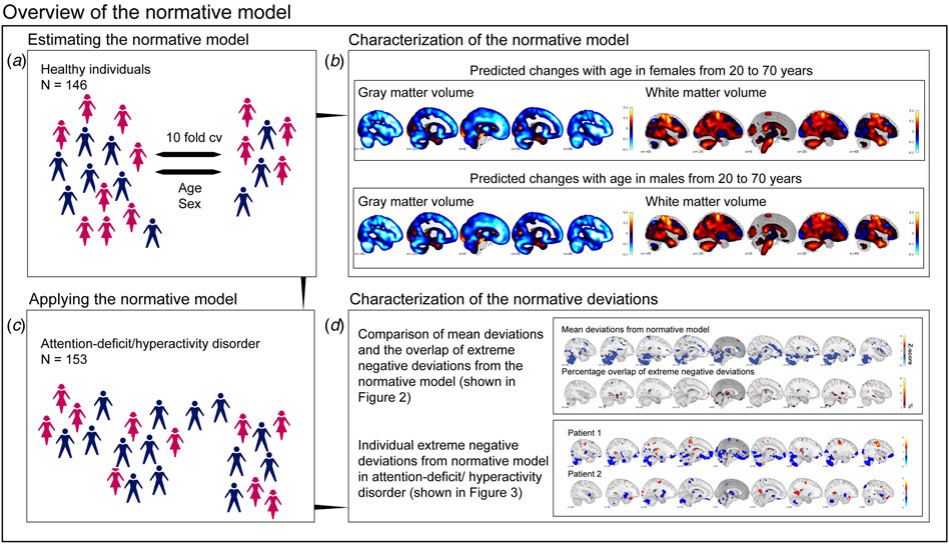
In (*a*), the estimation of the normative model in healthy individuals is depicted using age and gender as covariates. In (*b*), the characterization of the normative model is shown. We see that the normative model changes with age and that, from age 20 to 70 years, gray matter is predominantly decreasing; this is true for both females and males and more strongly observed in frontal brain regions. Blue colors indicate a decrease, red colors an increase. In (*c*), we depict the application of the normative model to persistent ADHD. In (*d*), we present the steps that were taken to characterize the deviations from the normative model.

The normative modeling method employed here is described in the supplemental methods (Marquand *et al*., 2016*a*). Briefly, normative models were estimated using Gaussian process regression (Rasmussen and Williams, 2006), a Bayesian non-parametric interpolation method that yields coherent measures of predictive confidence in addition to point estimates. This is important, as we used this uncertainty measure to quantify both the centiles of variation within the cohort and the deviation of each patient from the group mean at each specific brain locus. In this way, we were able to statistically quantify deviations from the normative model with regional specificity, by computing a *Z*-score for each voxel, reflecting the difference between the predicted volume and the true volume normalized by the uncertainty of the prediction (Marquand *et al*., 2016*a*). Thus, we quantified extreme positive and negative deviations (reflecting increased or decreased volume, respectively) from the normative model using a reasonable threshold for the resulting *z*-statistic. In the present study, we estimated normative brain changes across the adult lifespan represented in our study (Fig. 1) using Gaussian process regression to predict regional gray and white matter volumes across the brain from age and sex. The normative range for this model in healthy individuals was estimated using 10-fold cross-validation, then we applied the model trained on all healthy individuals to participants with ADHD.

First, we assessed group-level deviations from the normative model. For this, individual gray and white matter deviation maps were fed into PALM (Permutation Analysis of Linear Model; Winkler *et al*., 2015), which allowed for permutation-based inference. We estimated mean group-level deviations from the normative model in healthy individuals and in patients with ADHD. PALM creates a map of *z*-values for each of these groups. We thresholded these group maps using *Z* =  ±  2.6, to assist comparisons with the individual maps of deviation described below. Further, we report the contrasts for participants with persistent ADHD and healthy individuals corrected for false discovery rate (FDR) at the 5% inference level using threshold-free cluster enhancement.

Next, the individual maps of deviation were thresholded at |Z| > 2.6. These maps reflect the deviation from the normative model at the individual level. Note that the use of a fixed statistical threshold across participants allows for a simplified comparison between participants in terms of numbers of extreme deviation from the normative model, even when the overall distribution of deviations of a participant is shifted. We also repeated the analyses correcting for multiple comparisons at the individual participant level using the Benjamini and Hochberg procedure (Benjamini and Hochberg, 1995). This did not change our conclusions. Extreme positive deviations were defined as all voxels with a value higher than *Z* > 2.6, while extreme negative deviations are defined as a value below the *Z* < −2.6. All extreme deviations were combined into scores representing the percentage of extreme positive and extreme negative deviations for each participant. We tested for associations between diagnosis and those scores using a non-parametric χ^2^ test in a general linear model. We corrected for multiple comparisons using the Bonferroni-Holm method (Holm, 1979). We created individualized maps of extreme deviations and calculated the voxel-wise overlap between individuals from the same groups. In a final analysis, we tested for associations between the percentage of extremely deviating voxels and age, symptom scores, and comorbidity. We corrected for the number of correlations (8) and modality using the Bonferroni-Holm method (Holm, 1979). All analyses were performed in python3.6 (www.python.org).

## Results

### Participants

Table 1 shows the demographics of the study population. We included 153 adults with ADHD and 146 healthy adults. About the same proportion of individuals in both groups were male (43.8% first and 41.2% second group, respectively). The average age of participants was 35 years in both groups with an age distribution that was very similar (online Supplementary Fig. S1). Individuals with persistent ADHD showed higher scores than healthy individuals for hyperactivity-impulsivity (5.45 *v.* 0.63; *t* test: *p* < 0.01) as well as inattention (7.27 *v.* 0.55; *t* test: *p* < 0.01).

**Table 1.** Demographics and clinical characteristics

	Healthy individuals	Attention-deficit/ hyperactivity disorder^a^
Demographics		
Total (N)	146	153
Males (%)	43.8%	41.2%
Age (years) (mean ± std)	35.43 ± 12.01	35.05 ± 10.81
Education (years ± std)	5.19 ± 0.808	4.78 ± 0.811
Estimated intelligence^b^ (mean ± std)	109.94 ± 14.53	107.45 ± 15.08
Symptoms		
Hyperactivity/impulsivity^c^ (mean ± std)	0.63 ± 1.12	5.45 ± 2.46
Inattention^d^ (mean ± std)	0.55 ± 1.21	7.27 ± 1.74
Comorbidities^e^ (mean ± std)	0.01 ± 0.117	0.20 ± 0.436
Stimulant medication	Current = 0.0% Past = 0.0% No medication = 100.0%	Current = 11.8% Past = 76.4% No medication = 11.8%

a ADHD diagnosis was based on a structured Diagnostic Interview for ADHD in Adults (DIVA; Sandra Kooij *et al*., 2008).

b Estimated intelligence was based on the block-design and vocabulary subtests of the Wechsler Adult Intelligence Scale (WAIS-III; Wechsler, 2012).

c DIVA hyperactivity/impulsivity symptoms in adults.

d DIVA inattention symptoms in adults.

e Number of comorbid disorders such as major depressive disorder based on a SCID (Structured Clinical Interview) interview (van Groenestijn *et al*., 1999; Weertman *et al*., 2003; Lobbestael *et al*., 2011)

### Normative model

Figure 1*a*, *c* and *d* show a high-level visual summary of the analysis procedure. Figure 1*b* depicts a spatial representation of the voxel-wise normative model. This model was characterized by global gray matter decreases from age 20 to 70 years, with the largest decreases primarily in frontal and cerebellar regions, which is in line with the typical decline of gray matter volume over age (Ziegler *et al*., 2012; Farokhian *et al*., 2017). This was true for females and males, which we modeled separately due to the presence of sex effects in ADHD (Martin *et al*., 2018). In contrast, the normative model for the white matter was characterized by both decreases and increases across adulthood. More specifically, parietal and temporal brain regions showed an increase with age, areas in frontal and in particular thalamic regions showed decreased, in both sexes. This is in line with earlier reports on healthy aging (Farokhian *et al*., 2017). In online Supplementary Fig. S2, we depict the mean deviation of the normative model across all ages separately for females and males.

### Characterization of mean deviations from the normative model

**Fig. 2.**
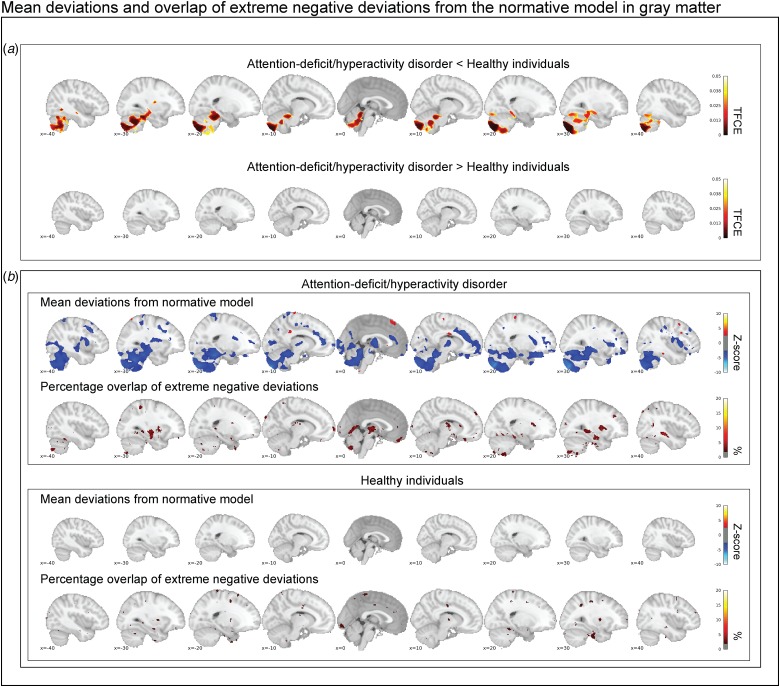
In (*a*), the contrast between persistent ADHD and healthy individuals is depicted corrected at a false discovery rate of 5%. Cerebellar regions, temporal regions, and the hippocampus deviate significantly in gray matter. In (*b*), the group-level mean deviations of participants with persistent ADHD and healthy individuals are depicted (|Z| < 2.6) and compared with the overlap maps of extreme negative deviations (*Z* < −2.6). In summary, while we reproduce prominent group-level differences between healthy individuals and participants with persistent ADHD, we observe that extreme negative deviations are hardly present in more than 2% of the individuals with persistent ADHD in those brain regions.

Figure 2*a* shows the mean deviations from the normative model in the gray matter for healthy individuals and those with ADHD. Individuals with ADHD and healthy individuals differed significantly after correction for multiple comparisons in their mean deviations from the normative model in the cerebellum, temporal brain regions, and the hippocampus. Participants with ADHD on average showed larger mean negative deviations in those regions. Looking at *Z*-score maps thresholded at ±2.6, this pattern was confirmed, and additional regions showing negative mean deviations were observed in the anterior cingulate, insula, and frontal cortex (Fig. 2*b*). No differences in mean deviations between patients and controls were observed in white matter (online supplementary Fig. S3*a*), although some positive and negative mean deviations exceeded the *z*-score threshold of ±2.6 in patients: for instance, temporal brain regions showed positive deviations, while frontal and parietal regions showed negative deviations (online Supplementary Fig. S3*b*).

### Association of extreme deviations from the normative model with persistent ADHD

An analysis of the total percentage of extreme negative deviations in gray matter across the groups showed that participants with persistent ADHD differed significantly from healthy individuals (Wald χ^2^(1) = 23.64, *p*_corr._ < 0.001). This effect was driven by a larger percentage of negative deviations in participants with persistent ADHD (0.48%; 95% confidence interval 0.30–0.66%) than in healthy individuals (0.28%; 95% confidence interval 0.24–0.34%). In white matter, significant differences in the percentage of extreme negative deviations were observed between groups as well (Wald χ^2^(1) = 18.02, *p*_corr._ < 0.001); again, a significantly higher proportion of negative deviations was seen in participants with persistent ADHD (0.41%; 95% confidence interval 0.24–0.57%) than in healthy individuals (0.24%; 95% confidence interval 0.17–0.31%). No differences between groups were observed in positive deviations on measures in gray and white matter (online Supplementary Table S1). As only the percentage of extreme negative deviations were significant between individuals with persistent ADHD and healthy participants we focus our characterizations of those deviations on extreme negative deviations in gray matter, we report the other extreme deviations in different modalities in the supplement but report the main outcomes in the section below as well.

### Characterization of extreme negative deviations from the normative model

**Fig. 3.**
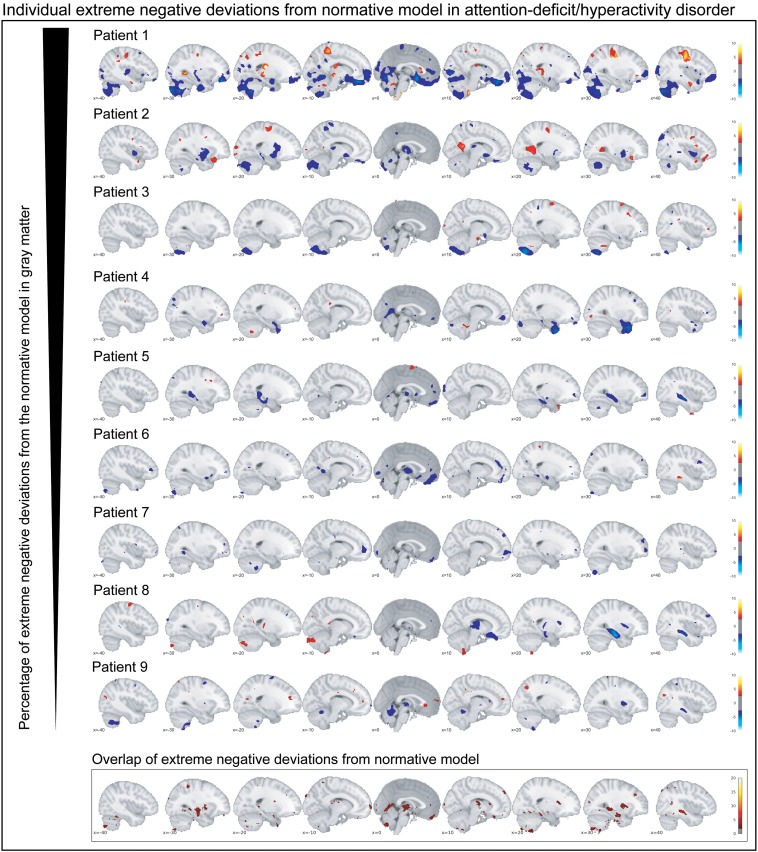
The individual extreme negative deviations from the normative model in gray matter are depicted for participants with persistent ADHD. Below the corresponding overlap map in gray matter is depicted. In summary, individual extreme deviations show a very unique pattern across participants with persistent ADHD.

Participants with ADHD showed overlap in local gray matter negative deviations in more than 2% of patients primarily in the cerebellum, hippocampus, and basal ganglia; less overlap in negative deviations was observed in healthy individuals (Fig. 2*b*). In white matter, we also observed greater overlap in participants with ADHD than in healthy individuals, again involving regions around the hippocampus and the basal ganglia (online supplementary Fig. S3*b*). A scattered pattern of positive deviations was seen in the (online supplementary Fig. S4) overlap maps for participants with ADHD as well as for healthy individuals in both gray and white matter. The overlap maps of the extreme negative deviations partly resembled the pattern observed in the mean deviation analyses of cases and controls (Fig. 2*b*), also when detecting extreme deviation based on the FDR (online Supplementary Fig. S5). Further, nine out of the ten most negatively deviating patients showed extreme values in the cerebellum (Fig. 3), although in non-overlapping areas. Generally, deviations in both positive and negative directions were unique for each participant with ADHD in gray and white matter, when looking at the patterns of individual deviations, with limited overlap (online Supplementary Fig. S6). The extreme negative deviations were associated with age in participants with ADHD (*β*-weight = 0.198, *p* = 0.014), but not symptom scores, stimulant medication, or comorbidity, before correction for multiple comparisons (online Supplementary Table S1); for the extreme positive deviations, we did not find any associations that were even nominally significant.

## Discussion

We mapped the biological heterogeneity of persistent ADHD in reference to normative brain aging across the adult lifespan, based on voxel-based morphometry derived brain measures. In participants with ADHD, we observed robust mean deviations in gray matter from the normative model in the cerebellum, temporal regions, and the hippocampus. However, at the individual level, we found that few brain loci showed extreme negative deviations in more than 2% of the participants with ADHD, providing a measure for the (substantial) inter-individual variation between adults with persistent ADHD.

Case-control comparisons show small to medium effect sizes of (gray matter) alterations in adult ADHD patients (Frodl and Skokauskas, 2012; Ziegler *et al*., 2012; Hoogman *et al*., 2017). Here, we show that some of these differences between participants with ADHD and healthy individuals in normative gray matter deviations are consistent with these earlier case-control findings. Note that our approach differs as we modeled the healthy range prior to computing group-level differences on the basis of deviations from normative aging. That said, mean normative differences in hippocampus and temporal region overlap with regions that have earlier been identified in children with ADHD (Hoogman *et al*., 2017). In addition, we observed mean normative deviations in the cerebellum; a decreased gray matter was seen in individuals with ADHD across the adult lifespan. The cerebellum is of increasing interest in ADHD (Berquin *et al*., 1998): for example, in case-control studies, those with ADHD have shown a decreased size of the cerebellum (Carmona *et al*., 2005; Ivanov *et al*., 2014), which may be linked to timing problems that are present across many individuals with this disorder (Aase and Sagvolden, 2005). We do not observe a robust difference in the prefrontal cortex or basal ganglia, regions that have often been implicated in (childhood) ADHD (Faraone and Biederman, 1998; Frodl and Skokauskas, 2012). However, when reducing thresholding in the group-level maps (|Z| > 2.6), these regions do present reductions in gray matter volume also in the current study (Fig. 2).

Whilst the group-level results based on normative deviations described above are largely in line with existing ADHD literature and point to the cerebellum as an important structure in persistent ADHD, we additionally observe a large biological heterogeneity at the level of the brain. Specifically, we found that only a few individual brain loci showed extreme negative deviations in more than 2% of the participants with ADHD, providing quantitative evidence of the biological heterogeneity of persistent ADHD (Faraone *et al*., 2015) and showing that inter-individual differences at the level of brain structure are a hallmark for this phenotype. This is consistent with conceptual developments such as the Research Domain Criteria (Insel *et al*., 2010), which emphasize the importance of moving beyond simple group comparisons in psychiatry towards multilevel, high-dimensional descriptions of individual patients. Our finding that patients with persistent ADHD differ substantially on an individual level speaks against the concept of the ‘average ADHD patient’ and suggests that it does not sufficiently reflect the degree of inter-individual variation that characterizes this disorder. This may explain why case-control studies, which dominate research on ADHD and mental disorders in general, have shown small group differences between patients and healthy individuals (Franke *et al*., 2009; Hamshere *et al*., 2013; Onnink *et al*., 2014; Faraone *et al*., 2015; Greven *et al*., 2015; Wolfers *et al*., 2015*b*, 2017; Francx *et al*., 2016). We expect a high degree of inter-individual differences for other biological readouts (e.g. functional measures) but quantifying the degree and mapping the nature of such heterogeneity is an important topic of future research.

Voxel-based morphometry studies are fundamentally reductionist, comparing group differences on the voxel by voxel level, making strong assumptions on (i) a single locus contributing to a disorder and (ii) group homogeneity. This approach has been extended by pattern classification studies, which consider multiple voxels at once and show that using structural MRI the predictions of ADHD range from about 60% to up to about 90% accuracy indicating a high variability between studies (Bansal *et al*., 2012; Igual *et al*., 2012; The ADHD 200 consortium, 2012; Lim *et al*., 2013; Peng *et al*., 2013; Johnston *et al*., 2014; Wolfers *et al*., 2015*a*). A prime example, the ADHD-200 competition, in which ADHD was predicted on the basis of different brain read-outs, showed predictions that did not exceed 60% accuracy (The ADHD 200 consortium, 2012). These outcomes were replicated in follow-up research, summarized in different reviews and studies using all kinds of brain imaging readouts (Sabuncu and Konukoglu, 2014; Wolfers *et al*., 2015*a*, 2016, 2017). Here, we used mass-univariate predictions, similar to voxel-based morphometry. However, unlike this approach we did not assume homogenous groups of individuals with ADHD and healthy participants. While this assumption is fundamental in voxel-based morphometry, it is also essential for pattern classification approaches. The present results question this assumption.

The present results allow for a novel interpretation of earlier large-scale pattern recognition studies in ADHD, which often showed relatively low accuracy in discriminating ADHD cases from controls (Wolfers *et al*., 2015*a*). In larger studies, the predictive accuracy for ADHD is reduced relative to smaller studies, which is counterintuitive to the premises of general machine learning, where an increase in sample size usually improves learning from data (Hastie *et al*., 2009). This conundrum can be understood in the context of the present results, as larger, more representative samples capture more of the biological as well as procedural heterogeneity (e.g. due to different scanners sites) of this disorder. Therefore, a larger sample will provide a better estimate of the variation between individuals. This increases the difficulty to find a common decision function across participants with ADHD in pattern classification analyses. Note, however, that larger studies also deal, to a greater extent with for instance acquisition inhomogeneities across different scanners, which might affect predictions negatively, while smaller studies may be more carefully controllable, or just by chance select a more homogenous subgroup.

We are confident that the present results and the main conclusions are replicable in follow-up studies. However, a few limitations require a discussion. First, we had to use 10-fold cross-validation in healthy individuals and out of sample predictions in individuals with persistent ADHD, as our healthy sample was too small to split it into two. This potentially introduces a small bias. Second, we did not find associations of symptom scores with the percentage of deviation from the normative model. However, the measures we used to assess symptoms rely on self-report, which is generally noisier than measures from diagnostic interviews. Finally, our sample did not allow to inspect the effect of comorbidities and other potentially confounding factors on the obtained results as the comorbidities were inconsistent across individuals. In future studies, we envision that normative models are built on the basis of large population samples. These population-based normative models can subsequently be applied to cohorts that sample ADHD using the same inclusion criteria for healthy individuals as for those with a disorder. In this way normative modeling is complementary to classical case-control comparisons as it allows for the investigation of individual differences. Here, we show that an approach relying on case-control differences is not sufficient to understand ADHD and its biological heterogeneity.

In conclusion, while our group level effects are largely in line with existing literature on ADHD, our approach also shows that the disorder is a much more biologically heterogeneous on the individual level than previously anticipated. We thus need to move towards descriptions of biology for the individual patient to improve our understanding of ADHD. The present results provide the first quantitative estimate of the degree of biological heterogeneity, in terms of spatial overlap of an individual's extreme gray and white matter deviations, linked to ADHD. In this way, we provide valuable information to improve the nosology and characterization of the different facets of persistent ADHD.

## Supplementary material

The supplementary material for this article can be found at https://doi.org/10.1017/S0033291719000084.
